# Molecular marker assisted gene stacking for biotic and abiotic stress resistance genes in an elite rice cultivar

**DOI:** 10.3389/fpls.2015.00698

**Published:** 2015-09-30

**Authors:** Gitishree Das, G. J. N. Rao

**Affiliations:** Biotechnology Laboratory, Crop Improvement Division, Central Rice Research InstituteCuttack, India

**Keywords:** rice, resistance genes, biotic stress, abiotic stress, molecular marker, gene stacking

## Abstract

Severe yield loss due to various biotic stresses like bacterial blight (BB), gall midge (insect) and Blast (disease) and abiotic stresses like submergence and salinity are a serious constraint to the rice productivity throughout the world. The most effective and reliable method of management of the stresses is the enhancement of host resistance, through an economical and environmentally friendly approach. Through the application of marker assisted selection (MAS) technique, the present study reports a successful pyramidization of genes/QTLs to confer resistance/tolerance to blast (*Pi2, Pi9*), gall Midge (*Gm1, Gm4*), submergence (*Sub1*), and salinity (*Saltol*) in a released rice variety CRMAS2621-7-1 as Improved Lalat which had already incorporated with three BB resistance genes *xa5, xa13*, and *Xa21 to* supplement the *Xa4* gene present in Improved Lalat. The molecular analysis revealed clear polymorphism between the donor and recipient parents for all the markers that are tagged to the target traits. The conventional backcross breeding approach was followed till BC_3_F_1_ generation and starting from BC_1_F_1_ onwards, marker assisted selection was employed at each step to monitor the transfer of the target alleles with molecular markers. The different BC_3_F_1_s having the target genes/QTLs were inter crossed to generate hybrids with all 10 stress resistance/tolerance genes/QTLs into a single plant/line. Homozygous plants for resistance/tolerance genes in different combinations were recovered. The BC_3_F_3_ lines were characterized for their agronomic and quality traits and promising progeny lines were selected. The SSR based background selection was done. Most of the gene pyramid lines showed a high degree of similarity to the recurrent parent for both morphological, grain quality traits and in SSR based background selection. Out of all the gene pyramids tested, two lines had all the 10 resistance/tolerance genes and showed adequate levels of resistance/tolerance against the five target stresses. The study demonstrates the potential of MAS for stacking of several genes into a single line with a high degree of parental genome recovery.

## Introduction

The rate of world population growth has exceeded the rate of growth in food-grain production. It is predicted that the world population will exceed 8 billion people by 2025 and to meet these global food demands, the production of grain needs to increase upto 50% more by the year 2025 (Khush, 1999). The emergence of new diseases and pests and the changing climate are the major issues that address the requirement for sustainable crop development and resistance to biotic and abiotic stresses (Hasan et al., 2015). Due to the sustained efforts by conventional breeding approaches, over the years, many significant progresses have been achieved in the development of suitable cultivars to effectively combat different types of the biotic and abiotic constraints that affect the productivity of rice. The occurrences of new biotypes/stresses have demanded stacking of several resistance genes into high yielding cultivar background to confer a wider spectrum of resistance. This enhanced capability will enable them to survive attacks from several pathogens at a time and also survive in unfavorable environmental conditions.

A rigorous yield loss that has affected the rice cultivation is accounted to biotic and abiotic stresses. In the recent times, technological innovations and development of DNA based molecular markers has facilitated the transfer of genes that confer resistance to different biotic stresses (BB, blast, and gall midge etc.) and abiotic stresses (Submergence and Salinity etc.). With the advances made in the area of molecular markers, the tracking of the genes for resistance is possible by following the path of markers that are linked/ tagged to each gene for resistance, thus making the identification of plants with two and more genes simple.

One of the most serious and widespread diseases in rice production is bacterial blight (BB) caused by *Xanthomonas oryzae pv.oryzae*. It reduces the yield of rice drastically by partial grain filling because of constraint to the photosynthetic area (Pradhan et al., 2015). There are no effective chemical agents against the bacterial blight pathogen. The only way to protect the crop from bacterial blight is the use of resistant varieties of rice (Khush et al., 1989). So far, more than 30 BB resistance genes have been identified and some of them have been incorporated into modern high yielding rice varieties (Dokku et al., 2013a,b; Suh et al., 2013; Kumar et al., 2014; Pradhan et al., 2015). In the presence of a number of virulent pathogens, the genes may differ in their level of resistance to them. Thus, gene stacking is currently being pursued in an effort to develop more durable and comprehensive resistance rice varieties to combat the effect of BB pathogens (Huang et al., 1997; Dokku et al., 2013a,b; Suh et al., 2013; Kumar et al., 2014; Pradhan et al., 2015).

The Rice blast disease occurs in most of the rice growing areas of the world (Ou, 1985). It is the most important fungal rice disease, which is caused by the fungus *Magnaporthe grisea* Barr. (Telomorph *Pyricularia oryzae* Sacc). Yield loss due to blast can be as high as 50%, when the disease occurs in epidemic proportions (Babujee and Gnanamanickham, 2000). In severe cases, yield losses can be 70–80% due to the fungus blast alone. In the wet season, due to encouraging environmental conditions for disease development, this disease arises frequently in the rice cultivars. For blast resistance, closely linked DNA markers to a blast R gene can be effectively used for marker assisted selection, which is comparatively faster than the conventional rice breeding methods (Singh et al., 2015). Till 2015, around 100 distinctive blast resistance genes have been identified. Out of them, minimum 14 number of genes (*Pi1, Pi2, Pi9, Pi20* (t), *Pi33, Pi39, Pi40* (t), *Pi47, Pi48, Pi54r*h, *Pi56, Piz, Piz-t*, and *Pigm*) have been defined as per their wide scale resistance (Hayashi et al., 2010; Huang et al., 2011; Das et al., 2012; Hua et al., 2012, 2015; Liu et al., 2013). The use of different resistant rice varieties in the cultivation of rice, could be the most active, and cost effective way to reduce the adverse effect of the blast disease. Thus, the improvement of resistant varieties against blast disease is one of the most important objectives in the rice breeding plan (Gouda et al., 2013; Divya et al., 2014).

The Asian rice gall midge, *Orseolia oryzae* is a serious pest of rice in certain regions of south, central, and east India, causing significant yield loss, mainly during the kharif season. Gall midge insect causes an annual yield loss of about 477,000 tons of grain or 0.8% of the total production with crop losses in the range of 10–100% in India. The estimate suggested an annual yield loss of US$80 million in India and of $550 million in Asian continent (Biradar et al., 2004). Till now, 11 gall midge resistance genes have been identified in plant and seven biotypes of the pest have been reported (Dutta et al., 2014; Hasan et al., 2015). For control of this pest, development of resistant rice varieties using marker assisted selection can be a sustainable and cost-effective approach (Dutta et al., 2014). Gene pyramiding with two or additional active genes in a single variety may lead to strong gall midge resistance rice varieties. Nowadays, the use of molecular markers for improvement of gene pyramids in desired combination is being monitored in different rice cultivars and presently using DNA markers for selection of resistant plants for gene pyramiding has been accepted as an established tool (Sundaram et al., 2008; Dutta et al., 2014).

Among the abiotic stresses, submergence is one of the vital issues in the flash flood prone rice cultivating areas (Iftekharuddaula et al., 2015). Submergence tolerance is an important trait for rice (*Oryza sativa*) in rain-fed lowland conditions. This trait is largely controlled by a major gene designated as *Sub1*. Indica cultivar FR13A, is a highly tolerant rice variety which can survive upto 2 weeks of complete submergence owing to a major quantitative trait locus designated as submergence1 (Sub1) near the centromere of chromosome 9 (Xu and Mackill, 1996; Xu et al., 2000; Chen et al., 2002; Septiningsih et al., 2013; Manivong et al., 2014). For submergence tolerance the background genetic information was well documented from a number of researches using QTL mapping and map based cloning approaches (Septiningsih et al., 2009; Manivong et al., 2014; Singh et al., 2014a).

Salt stress is a major constraint across many rice producing areas because of the high sensitivity of modern rice varieties. It is one of the top most abiotic stresses that, imposes a limitation to the growth and improvement of rice plant, triggering yield losses of more than 50% (Molla et al., 2015). Tolerance to salinity is complex, involving a number of different physiological mechanisms, such as sodium exclusion from roots, controlled sodium transport between root and shoot, and sequestering of sodium in older tissues and in the vacuoles. It has been estimated that over 150 million hectares of current and potential rice land in the tropical and subtropical regions of the world are affected by salinity (Dissanayake and Wijeratne, 2006; Molla et al., 2015). Even though, rice is the major food source for half of the world population, comparatively it is more susceptible to salt stress than other cereals (Vu et al., 2012).

To stabilize the biotic and abiotic stresses in rice, the improvement of resistant variety can be considered to be the utmost effective technique. As the cultivars undergo quick collapse in their resistance, there is a requirement of the development of highly resistant varieties. Thus, bringing together several genes conferring resistance to more than one stress into a single genetic background is necessary for its durable resistance (Singh et al., 2014b).

Hence, the main objective of the present study is pyramidization/stacking of genes/QTLs conferring resistance/tolerance to blast (*Pi2, Pi9*), gall midge (*Gm1, Gm4*), submergence (*Sub1*), and salinity (*Saltol*) in Improved Lalat (*Xa4, xa5, xa13, and Xa21)*, an elite released rice variety.

## Materials and methods

### Plant material and breeding strategy

**Figure 1:**
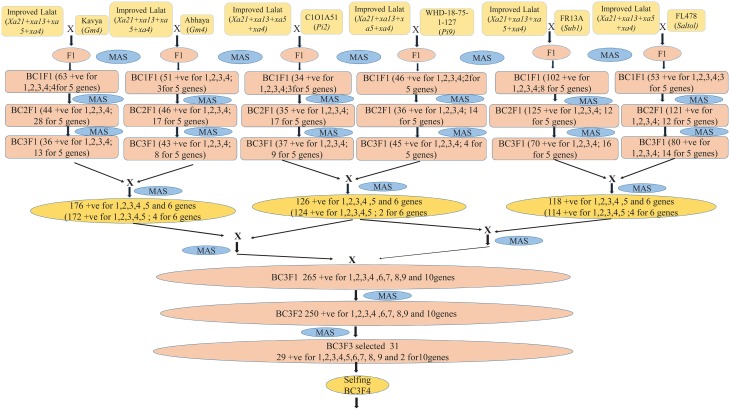
**A flow diagram depicting the development of Improved Lalat pyramids through gene stacking different steps**.

A well-adapted *indica* rice genotype Improved Lalat having four bacterial blight resistance genes (*Xa4, Xa21, xa13*, and *xa5*) developed in CRRI (CRRI, India, Annual Report 2011-12^1^; Dokku et al., 2013a) was chosen as recurrent parent. Two cultivars, C1O1A51 (Deng et al., 2006) and WHD-1S-75-1-127 *(O. minuta* derivative) with blast resistance genes (*Pi2 and Pi9* respectively) (Hittalmani et al., 2000; Singh et al., 2011), two cultivars, Kavya and Abhaya with Gall midge resistance genes (*Gm1 and Gm4* respectively) (Kumar et al., 2000; Biradar et al., 2004; Kumaravadivel et al., 2006; Himabindu et al., 2010), one cultivar, FR13A (Xu and Mackill, 1996; Nandi et al., 1997; Xu et al., 2000; Septiningsih et al., 2009) with submergence resistance QTL (*Sub1*) and one cultivar FL478 with salinity resistance QTL (*Saltol*) genes were chosen as the donor parents (Bonilla et al., 2002; Nejad et al., 2008, 2010). The DNA markers linked to BB, blast, gall midge resistance and submergence, and salinity tolerance were based on published literature (Table 1). The present study has employed the molecular markers available for resistance genes/QTLs like *xa5, xa13, Xa21 (BB)* (Shanti et al., 2010; Singh et al., 2011; Dokku et al., 2013a,b; Suh et al., 2013; Kumar et al., 2014; Pradhan et al., 2015), *Pi2, Pi9* (Blast) (Wang et al., 1994; Hittalmani et al., 1995; Hayashi et al., 2010; Huang et al., 2011; Singh et al., 2011; Das et al., 2012; Hua et al., 2012, 2015; Liu et al., 2013; Jiang et al., 2015), *Gm1, Gm4* (Gall Midge) (Mohan et al., 1994; Nair et al., 1996; Biradar et al., 2004; Himabindu et al., 2010; Rawat et al., 2012; Dutta et al., 2014), Sub1 (Submergence) (Xu et al., 2006; Manivong et al., 2014; Hasan et al., 2015; Iftekharuddaula et al., 2015), and *Saltol* (Salinity) (Nejad et al., 2008, 2010; Singh et al., 2011; Vu et al., 2012; Hasan et al., 2015) (Table 1) for gene pyramiding in to Improved Lalat an elite *Indica* genotype that have a wide coverage to present a multiple gene barrier (along with *Xa4, xa5, xa13, and Xa21*) against several stresses like bacterial blight, blast, gall midge, submergence and salinity in rice in the minimum number of generations with high precision through an efficient foreground (MAS for the gene of interest) and background selection (MAS for recovery of the recurrent parental genome). To achieve the objectives, the parent was hybridized and marker assisted back cross breeding approach was practiced in each generation (Figure 1).

**Table 1:** **Linked markers used for foreground selection of BB, Blast, Gall midge, Submergence, Salinity resistance/tolerance genes, their chromosome location, and primer sequence**.

**Diseases/Stresss**	**Linked genes**	**Markers**	**Ch. no**.	**Primer sequence**	**References**
Bacterial blight	*xa13*	Xa13prom	8	GGCCATGGCTCAGTGTTTAT GAGCTCCAGCTCTCCAAATG	Zhang et al., 1996; Singh et al., 2011; Dokku et al., 2013b
	*Xa21*	pTA 248	11	AGACGCGGAAGGGTGGTTCCCGGA AGACCGGTAATCGAAAGATGAAA	Ronald et al., 1992; Dokku et al., 2013b
	*xa5*	RG556	5	TAG CTG CTG CCG TGC TGT GC AAT ATT TCA GTG TGC ATC TC	Yoshimura et al., 1995; Dokku et al., 2013b
Blast	*Pi2*	RG64	6	CTGCAGTGCAATGTACGGCCAGG CTGCAGTGCAATGTACGGCCAGG	Hittalmani et al., 1995; Liu et al., 2002
	*Pi9*	P28	6	TGCTGACTGCTTGCTATTCGT GT ACTTCATCTTGAGCGACGCAA	Qu et al., 2006
Gallmidge	*Gm1*	RM444	9	GCTCCACCTGCTTAAGCATC TGAAGACCATGTTCTGCAGG	Biradar et al., 2004
	*Gm4*	RM547	8	TAGGTTGGCAGACCTTTTCG GTCAAGATCATCCTCGTAGCG	Nair et al., 1996
Submergence	*Sub1*	SUB1BC2	9	AAAACAATGGTTCCATACGAGAC GCCTATCAATGCGTGCTCTT	Xu et al., 2006
Salinity	*Saltol*	RM10745	1	TGACGAATTGACACACCGAGTACG ACTTCACCGTCGGCAACATGG	Bonilla et al., 2002; Nejad et al., 2008, 2010

### Parental polymorphism survey with the markers linked to gall midge, blast, submergence, and salinity resistance/tolerance genes/QTLs

**Figure 2:**
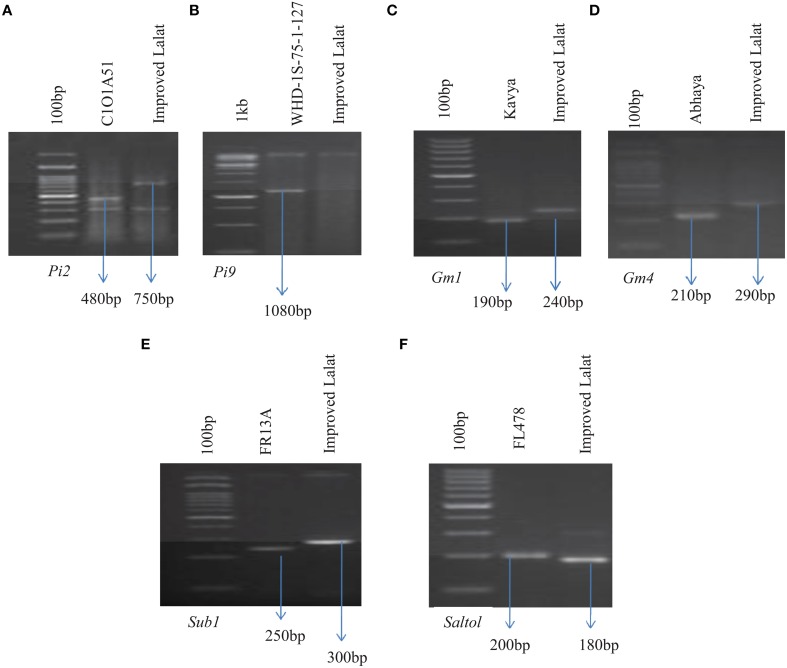
**Parental polymorphism assay by PCR analysis of parental lines. (A)** Amplification with RG64 (digested with *HaeIII*) linked to *Pi2* (C1O1A51). **(B)** Amplification with P28 linked to *Pi9* (WHD-1S-75-1-127). **(C)** Amplification with RM444 linked to *Gm1*(KAVYA). **(D)** Amplification with RM547 linked to *Gm4* (ABHAYA). **(E)** Amplification with SUB1BC2 linked to *Sub1* (FR13A). **(F)** Amplification with RM10745 linked to *Saltol* (FL478). Arrows indicate polymorphic bands that are linked to each respective resistance gene.

A parental polymorphism survey was conducted with the recurrent parent involved, i.e., Improved Lalat, and the donor parents, i.e., Kavya (Carrying the gall midge resistance gene *Gm1*), Abhaya (Carrying the gall midge resistance gene *Gm4*), C1O1A51 (Carrying the blast resistance gene *Pi2*), WHD-1S-75-1-127 (Carrying blast resistance gene *Pi9*), FR13A (Carrying submergence resistance QTL *Sub1*), and FL478 (Carrying salinity resistance QTL *Saltol*) using the markers available in the public domain of the six different genes/QTLs. Presence of polymorphism was studied between the donors and the recurrent parents for markers linked to different genes/QTLs like RM444 (*Gm1*), RM547 (*Gm4*), RG64 (*Pi2*), P28 (*Pi9*), SUB1BC2 (*Sub1*), and RM10745 (*Saltol*) to relate the resistance and susceptibility associated with the markers in these genotypes (Figure 2).

### DNA isolation and PCR analysis for forward selection

Generally the population size in a breeding program is quite large DNA isolation for PCR analysis can be a limiting step in marker assisted selection (MAS). A technically simple, quick, and reproducible DNA isolation technique was carried out following the standard procedure described by Dellaporta et al. (1983). Leaf sample of (2 cm) was collected from 2 weeks old rice seedlings. The leaf tissue was cut into 0.3 cm and placed in a well on the spot test plate (Thomas Scientific). The CTAB extraction buffer was added and grounded with a thick glass rod. The samples were transferred to the 1.5 ml Eppendorf tube and equal volume of chloroform was added to it and mixed well centrifuged at 17°C at 12,000 rpm for 5 min. The top aqueous phase was transferred to another 1.5 ml Eppendorf tube, to which two times volume of ethanol (99.9%) was added and mixed well. Furthermore, it was centrifuged at 10,000 rpm for 3 min at 4°C and the supernatant was decanted. The pellets were washed with 70% ethanol and the DNA was air dried and resuspended in 50 μl of 0.1X TE and stored at −20°C. The quantification of DNA was accomplished by using UV-VIS spectrophotometer (UV-1201 Schimazu Corp; Japan).

For foreground selection, sequence-tagged site (STS) and microsatellite (SSR) markers were used for all the biotic and abiotic stress resistance genes. For BB resistance genes RG556 (Yoshimura et al., 1995), xa13prom (Singh et al., 2011), pTA248 (Ronald et al., 1992; Chunwongse et al., 1993) were used for *xa5, xa13, Xa21* respectively, RG64 (Hittalmani et al., 1995), P28 (Yu et al., 1991; Amante-Bordeos et al., 1992; Pan et al., 1996; Liu et al., 2002; Wu et al., 2005) were used for blast resistance genes *Pi2, Pi9* respectively, for gall midge resistance genes RM444 (Jain et al., 2004; Himabindu et al., 2010), RM547 (Jain et al., 2004; Himabindu et al., 2010) were used for screening of *Gm1, Gm4* respectively. For submergence, SUB1BC2 (Septiningsih et al., 2009) was used for *Sub1* QTL screening and for salinity, RM10745 (Nejad et al., 2008, 2010) was used for *Saltol* QTL screening. Above markers were tightly linked to each resistance gene/QTLs, *xa5, xa13, Xa21, Pi2, Pi9, Gm1, Gm4, Sub1*, and *Saltol* respectively, and were used to confirm the presence of each gene/QTL through PCR analysis.

The PCR reaction mixture contained 50 ng template DNA, 5 pico M of each of forward and reverse primers, 200 μM dNTPs, 1 X PCR buffer (10 mM Tris-HCl, pH 8.3, 50 mM KCl, 1.5 mM of MgCl_2_ and 0.01 mg/ml gelatin) and 0.5 U of Taq DNA polymerase (DreamTaq DNA Polymerase, Thermo Scientific) in a volume of 20 μl. Amplification cycling was performed in an EP gradient 96 V programmable master cycler (Eppendorf AG, Hamburg, Germany). For *xa5*, restriction enzyme *DraI* and for *Pi2*, restriction enzyme *HaeIII* was used. The final PCR products and DNA fragments produced by restriction digestion were resolved by electrophoresis on agarose gels and analyzed on the alpha imager (Alpha Innotech, USA).

### Screening against the bacterial blight pathogen

The bacterial blight (BB) isolates of the pathogen available at CRRI, India was used to screen pyramided lines of both genotypes under natural conditions in the field. For testing the reaction of pathogen, the top leaves of the plants were clip-inoculated using the clip inoculation method with the bacterial suspension at a density of 10^9^ cells/ml at maximum tillering stage (Kauffman et al., 1973). For evaluating the resistance of gene pyramids developed in this study, the isolate produced an average lesion length ranging from 6.0 to 18 cm in susceptible differentials and an average lesion length of 0.1–4.8 cm in the resistant differentials. The pyramids were planted in the field with spacing of 15 cm × 20 cm (between plants and between rows) nine leaves of three different plants (3 leaves per plant) in each of the tested lines were clip-inoculated and the phenotypic reactions of the lines were recorded 20 days after inoculation both by visual scoring and measurement of lesion length (LL). The distinction between resistant and susceptible plants was set at LL of 5 cm. Plants with LL of < 5 cm were scored as resistant and those with > 5 cm were scored as susceptible with slight changes (Dokku et al., 2013a,b). The screening was done with three sets of plants for each line and each set contains 18 plants.

### Screening procedure against blast pathogen

For screening against blast, the lay-out adopted was a Uniform Blast Nursery (UBN) pattern where each test entry (gene pyramid, parent etc.) are sown in a single row of 50 cm length and successive rows are 10 cm apart. After every 20 test entries, B 40, a susceptible variety was sown. The entire nursery was surrounded all sides by two rows of HR12, a well-known susceptible variety for the spread of the disease in the experiment. High doses of nitrogen (100–120 kg N/ha) were applied.

#### Artificial inoculation

The nursery was sown during the blast favorable weather conditions to facilitate infections and polycyclic development of the disease. To create severe blast incidence, additional inoculum was provided by collecting diseased leaves, chopping them into small pieces and scattering them over the nursery. In addition, infected plants are also being transplanted between border rows. This operation can also carried out during prolonged wet weather. The scoring was based on leaf blast severity on the SES scale and at least two readings on blast severity are recorded at 10 day intervals from 25 to 30 days (Roumen et al., 1997; IRRI, 2002). The screening was done with three sets of plants for each line and each set contains 18 plants.

### Screening procedure against gall midge

Screening for gall midge resistance was done by following the standard protocol (Vijaya Lakshmi et al., 2006). Ten days old seedlings of all the homozygous lines of Improved Lalat were grown in plastic trays along with resistant controls, i.e., Kavya, Abhaya, and a susceptible control such as T (N) 1. The trays were kept in cages and larvae of gall midge were released. After 20 days, the plants were scored for their reaction to gall midge and the appearance and percentage of galls (silver shoot) was recorded. Seedlings were scored for reaction in terms of percent plant damage. The plants were evaluated when the susceptible plants showed 90–100% plant damage with the appearance of gall. Plants without the appearance of gulls were cut up to detect dead maggots and for the existence of tissue necrosis as an indicator of oversensitive response to confirm as resistant lines. Test seedlings with 0–20% of damage were considered as resistant, whereas others were considered as susceptible. The screening was done with three sets of plants for each line and each set contains 18 plants.

### Screening procedure for submergence tolerance

The 21 days old seedlings from the pyramid lines of Improved Lalat were grown in plastic trays along with resistant controls (FR13A, IR64sub1) and susceptible controls (IR42 and Improved Lalat) were placed in submergence tanks of CRRI, India. The tanks were slowly filled with water, without disturbing the plants. The trays were at the bottom of the tank and the tanks were completely filled with 1.6 m standing water over the top of the leaves. After 15 days of complete submergence, all the water was drained from the tank. The trays were kept in the open air for 8 days (desubmergence) and the scoring for a survival rate percentage (%) was determined after 8 days of desubmergence (Jantaboon et al., 2011). The screening was done with three sets of plants for each line and each set contains 18 plants.

### Screening procedure for salinity tolerance

The salinity screening was carried out following the standard procedure of Fageria (1985) in the Central Rice Research Institute, India, experimental salinity tanks. These tanks are specifically designed for salinity screening. These tanks were filled with soil, and fertilizer was applied to it. A water tank was connected to the salinity tanks for water supply to the experimental tank. The 21 days seedlings of the pyramid lines of Improved Lalat were transplanted in the salinity tanks along with resistant controls, i.e., FL478, SR26B and susceptible controls, i.e., IR29, Improved Lalat (Recurrent parent). At the beginning 2 days the water tank was filled with salt water of EC (Electrical conductivity) 2 dS m^−1^ and the salt water was supplied to the experimental tanks. Up to 10 days the experimental tanks were maintained to (8–10 dS m^−1^) EC. The E.C. of soil and water was checked every day using conductivity meter. After 10 days, the salt concentration of soil was increased to E.C. 16 dS m^−1^. After 60 days, when the susceptible controls were completely died, the pyramids were scored for their survival rates. The survived plants kept in the same condition up to the reproductive stage. The spikelet fertility (%) was recorded during the reproductive stage. The screening was done with three sets of plants for each line and each set contains 18 plants.

### Characterization for agronomic performance and grain quality

The thirty days old selected promising pyramided lines of Improved Lalat with the parent Improved Lalat were transplanted with 15 × 20 cm spacing in a randomized complete block design with three replications at the experimental farm of the Central Rice Research Institute, India. Data were recorded from 10 plants of each genotype for agronomic traits like days to 50% flowering (DFF) was recorded in the days when about 50% of the tillers in the line exhibited panicle emergence. Plant height (PH) was measured in cm from the base of the hill to the tip of the tallest panicle on the 20th day after flowering, ear bearing tiller (EBT) was recorded as the number of panicles per hill. Panicle length (PL) was measured in cm from the ciliate base of the panicle to the tip of the top most spikelet. Grain number (GN) was recorded as the average number of grains of 10 random panicles, one each of the 10 sample hills. Grain weight (GW) was computed in grams as the weight of 1000- well filled oven dried grains at 13% grain moisture content.

The grain and cooking quality were analyzed from the harvested grain. Physico-chemical characters such as hulling, milling and head rice recovery percentage were determined as per the method of Ghosh et al. (1971). Head rice recovery (HRR) of the polished kernels is then passed through rice grader having different (mm) grooves. The whole grains were then separated from the broken grains in order to quantify the head rice recovery. Head rice recovery is the percentage of full length intact kernels after milling. This was calculated as (Weight of whole, polished kernels/Weight of paddy) × 100. Kernel length (KL) was the average of the length of 10 unbroken milled rice measured in mm by dial micrometer. Kernel breadth (KB) was the average of the breadth of 10 unbroken milled rice measured in mm by dial micrometer. L/B ratio was recorded as the length and breadth ratio of the kernel averaged over 10 unbroken kernels. Cooking qualities- 5 gm of whole milled rice was cooked for 20 min after pre-soaking in 15 ml water for 5 min. Kernel length after cooking (KLAC) was the average of the length of 10 cooked kernels measured in mm, elongation ratio (VER) was the average ratio of the length of cooked rice to kernel length of milled rice of 10 kernels. Volume expansion, for assessing volume expansion the method of Verghese (1950) was followed, Alkali spreading value (ASV), gelatinization temperature is estimated indirectly by the alkali digestion test (Little et al., 1958). Amylose content, amylose was determined following the method described by Juliano (1971).

### Statistical analyses

The scoring of amplified bands was done as present (1) or absent (0) for each marker allele-genotype combination. The data entry was done into a binary data matrix as discrete variables. The molecular weight of the bands was estimated using 100 bp DNA ladder as standard. Bands with the same molecular weight and mobility were treated as identical fragments. The total number of bands, distribution of bands across accessions, number of polymorphic bands in a set of accessions, and average number of bands per primer were calculated. The molecular data was analyzed using NTSYS-PC (Numerical Taxonomy and Multivariate Analysis System) computer package (Rohlf, 1990). The genetic similarity between accessions was calculated by Dice (SSR) and Jaccard's (RAPD) similarity coefficient and Euclidian distance coefficient for morphological data.

The term polymorphism information content (PIC) refers to the value of a marker for detecting polymorphism within a population, depending on the number of detectable alleles and the distribution of their frequency. In the present study, the PIC value of a marker was calculated according to Anderson et al. (1993). Dendrograms were constructed based on Sequential Agglomerative Hierarchical Nesting (SAHN) based Unweight Pair Group Method with Arithmetic Means (UPGMA) using the software package NTSYS PC 2.01, to infer genetic relationships and phylogeny. The dendrogram obtained was then used for cluster analysis.

## Results

### Pyramiding of blast, gall midge, submergence, and salinity resistance/tolerance genes

The molecular markers used in this study for foreground selection were, according to the published literature (Table 1). Marker assisted backcross breeding approach was practiced and foreground selection was done to select the plants having resistance alleles of all the targeted genes. Only the positive plants having resistance alleles was advanced to the next generation at each stage till BC_3_F_1_ generation (Figure 1). Clear polymorphism was observed with all the markers linked to the genes/QTLs under the study between the parents and the donors (Figure 2). The polymorphism was also validated by the phenotyping studies conducted against each stress.

The BC_3_F_1_ generation hybrids were inter crossed in different combinations so as to stack all the 10 genes together into a single genotype. The combinations attempted are [Improved Lalat + *Gm1*] × [Improved Lalat + *Gm4*], [Improved Lalat + *Pi2*] × [Improved Lalat + *Pi9*], and [Improved Lalat + *Sub1*] × [Improved Lalat + *Saltol*]. Resulting four plants with *Xa21, xa13, xa5, Xa4* + *Sub1* + *Saltol* gene combination, four plants with *Xa21, xa13, xa5, Xa4*+*Gm1*+*Gm4* gene combination and two plants with *Xa21, xa13, xa5, Xa4*+*Pi2*+*Pi9* gene combination (Figure 1).

**Figure 3:**
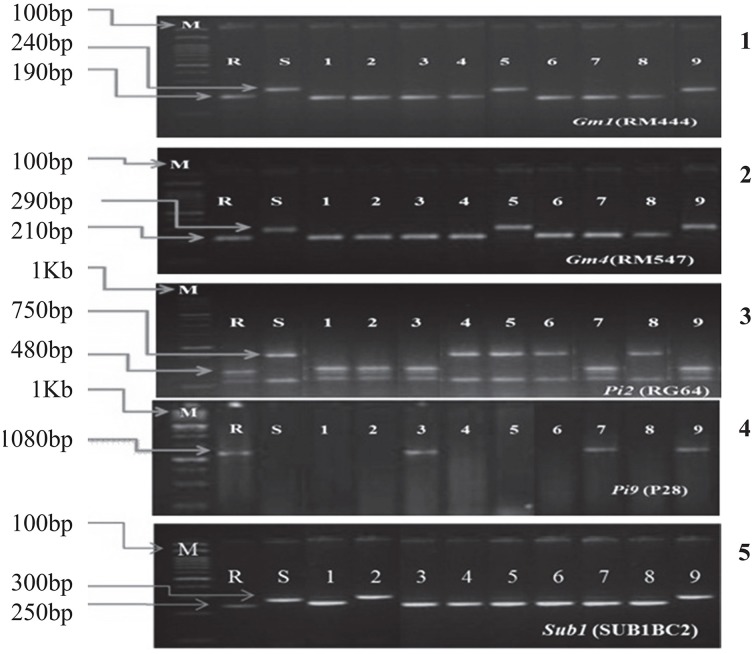
**PCR analysis of BC_3_F_3_ lines for the presence of resistant allele(s) of different genes/QTLs**. 1. DNA amplification of *Gm1* alleles using the primer RM444. 2. DNA amplification of *Gm4* alleles using the primer RM547. 3. DNA amplification of *Pi2* alleles using the primer RG64 and digested with *HaeIII*. 4. DNA amplification of *Pi9* allele using the primer P28. 5. DNA amplification of *Sub1* allele using the primer SUB1BC2. M = Marker. For *Gm1, Gm4*, and *Sub1* the marker was100 bp and for *Pi2* and *Pi9* the marker was 1 kb. S = Recurrent parent (susceptible) R = Resistant parent. The numbers (1–9) indicated in the Figure were gene pyramids of different gene combinations; 1 = ILGP1, 2 = ILGP3, 3 = ILGP5, 4 = ILGP12, 5 = ILGP13, 6 = ILGP14, 7 = ILGP19, 8 = ILGP20, 9 = ILGP17.

**Figure 4:**
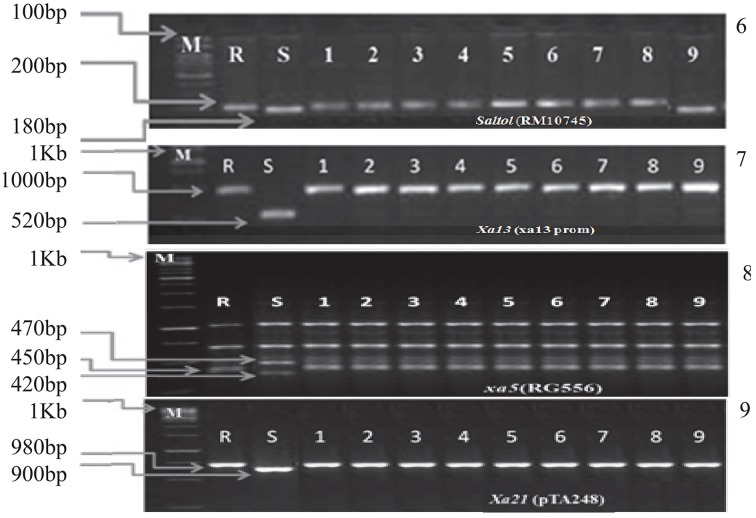
**PCR analysis of BC_3_F_3_ lines for the presence of resistant allele(s) of different genes/QTLs**. 6. DNA amplification of *Saltol* alleles using the primer RM10745. 7. DNA amplification of *xa5* alleles using the primer RG556 and digested with *DraI*. 8. DNA amplification of *Xa21* alleles using the primer pTA248. 9. DNA amplification of *xa13* alleles using the primer xa13Prom. M = Marker. For *Saltol* the marker was100 bp and for *Xa21, xa13*, and *xa5* the marker was 1 kb. S = Recurrent parent (susceptible) R = Resistant parent. The numbers (1–9) indicated in the Figure were gene pyramids 1 = ILGP1, 2 = ILGP3, 3 = ILGP5, 4 = ILGP12, 5 = ILGP13, 6 = ILGP14, 7 = ILGP19, 8 = ILGP20, 9 = ILGP17.

These hybrids were intercrossed in different combinations for two more cycles. The BC_3_F_3_ generation plants homozygous for five, six, seven, eight, nine, and ten genes were selected in resulting combination (Figure 1). From BC_3_F_3_ generation 31 pyramid lines with different gene combination were selected and nine lines were advanced for bioassay and background selection. The study employed three backcrosses to transfer the desired traits from the donors into Improved Lalat followed by three cycles of selfing. Through this approach, we could successfully transfer all the target genes/QTLs of resistance/tolerance into the Improved Lalat background using marker assisted selection in each step starting from BC_1_F_1_ generation. Of the two hundred fifty BC_3_F_3_ generation lines of Improved Lalat, plants homozygous for alleles associated with the resistance/tolerance were identified through marker analysis (Figures 3, 4).

### Bioassay of the gene pyramids for biotic and abiotic stresses

**Figure 5:**
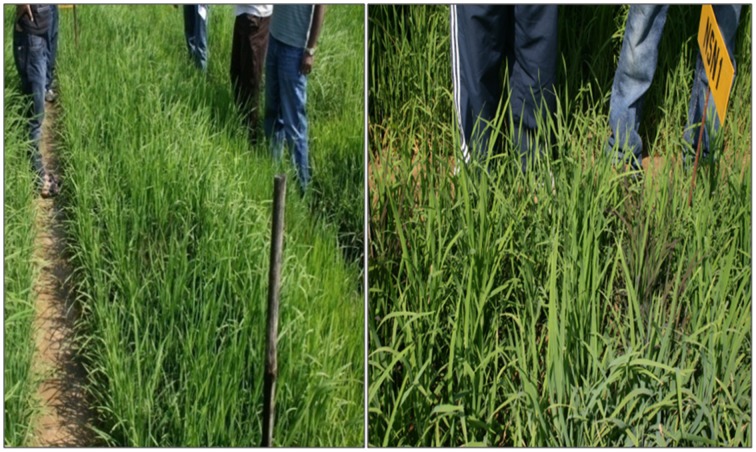
**Screening of Improved Lalat gene pyramids against Blast in BC_3_F_3_ generation (crops in the field)**.

**Figure 6:**
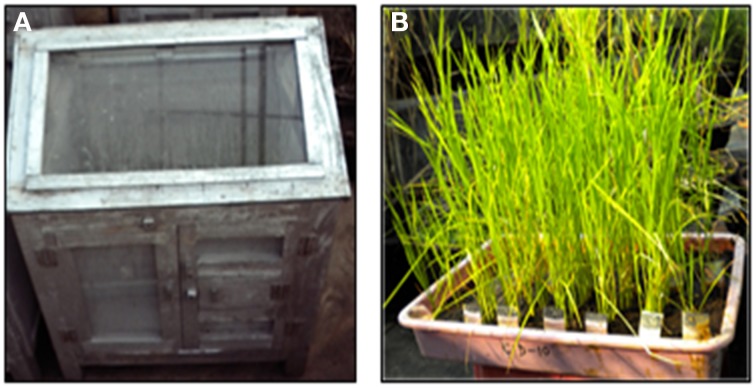
**Screening for resistance against gall midge (A) Cage (B) Tray containing IL pyramids**.

The bioassays confirmed the earlier finding that the recurrent parent, i.e., Improved Lalat having *Xa4, xa5, xa13, Xa21* incorporated in an earlier study possess high levels of resistance against BB. All the pyramided lines including the parent, the lesion length was below 3.4 cm (Table 2). The screening result against blast, the donors C1O1A51 and WHD-1S-75-1-127 were very effective against blast and showed resistant reaction (R) with a score of 0 while the recurrent parent showed a score of 4 showing susceptible (S) reaction. The gene pyramids ILGP5, ILGP17, and ILGP 19 having both the blast resistance genes *Pi2* and *Pi9* showed a high degree of resistance against blast disease (Table 3, Figure 5). The pyramid lines when screened against gall midge, the donor parents Kavya and Abhaya showed 100% resistance while the recurrent parents showed high susceptibility with 6.4% resistance and the pyramid lines showed resistance to gall midge ranging from 50 to 100% (Table 4, Figure 6).

**Table 2:** **Disease reaction of different Improved Lalat pyramid lines in BC_3_F_3_ generation against biotic stress Bacterial blight (*Xanthomonas oryzae*)**.

**Improved Lalat gene pyramids**	**Mean lesion length (in cm)**
IL-P	0.37 ± 0.15
ILGP1	0.72 ± 0.39
ILGP3	1.10 ± 0.42
ILGP5	2.00 ± 0.91
ILGP12	3.20 ± 2.61
ILGP13	2.30 ± 0.53
ILGP14	0.87 ± 0.47
ILGP17	1.37 ± 0.62
ILGP19	2.62 ± 1.10
ILGP20	3.37 ± 1.31
IRBB60	2.25 ± 1.25

*All results are expressed in mean ± SD. Values ≤ 5.00 cm was scored as resistant and values >5.00 cm were scored as susceptible, ILP, Improved Lalat parent*.

**Table 3:** **Disease reaction of different Improved Lalat pyramid lines in BC_3_F_3_ generation against biotic stresses blast**.

**Improved Lalat gene pyramids**	**Score**	**Reaction**
IL-P (S)	4	S
ILGP1	4	S
ILGP3	3	MR
ILGP5	0	R
ILGP12	4	S
ILGP13	4	S
ILGP14	4	S
ILGP 17	0	R
ILGP 19	0	R
ILGP 20	4	S
HR12 (S)	4	S
C1O1A51(R)	0	R
WHD-1S-75-1-127(R)	0	R

*ILP, Improved Lalat parent; R, Resistant; MR, Moderately Resistant; S, Susceptible; HR12 (S), Susceptible control; C1O1A51(R), resistant donor parent*.

**Table 4:** **Disease reaction to biotic stresses of different Improved Lalat pyramids in BC_3_F_3_generation against gall midge**.

**Improved Lalat gene pyramids**	**% of positive plants**
IL P (S)	6.40
ILGP1	75.00
ILGP3	83.30
ILGP5	100
ILGP12	83.30
ILGP13	58.33
ILGP14	94.10
ILGP 17	50.00
ILGP19	87.50
ILGP20	81.20
KAVYA (R)	100
ABHAYA (R)	100
TN1 (S)	0

*ILP, Improved Lalat parent; R, Resistant; S, Susceptible*.

**Figure 7:**
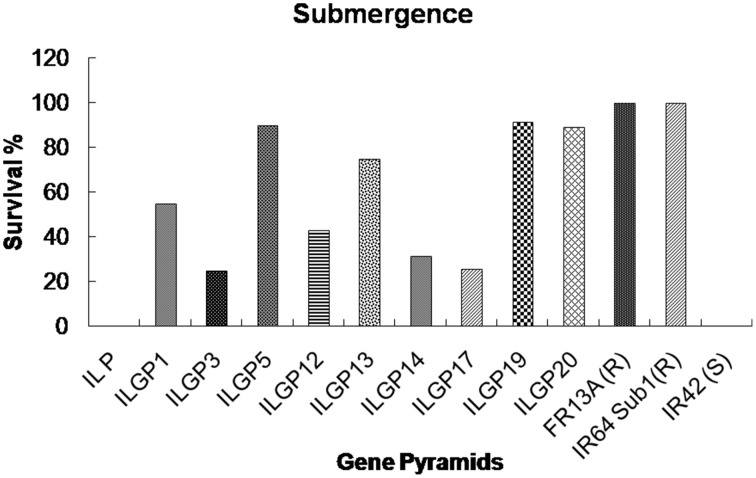
**Survival rates (%) of different Improved Lalat gene pyramid lines after 15 days of submergence stress**.

**Figure 8:**
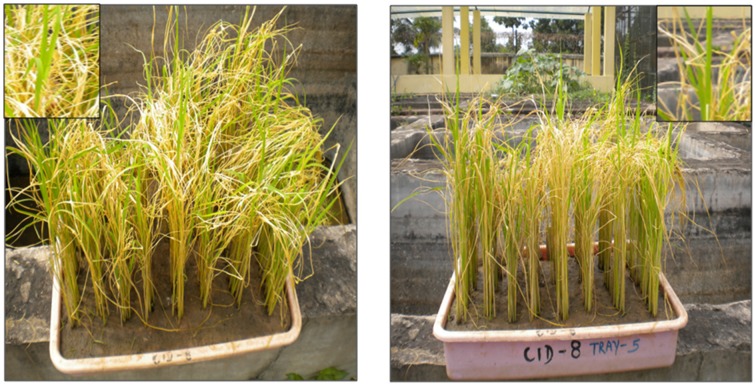
**Gene pyramids after 15 days of submergence stress and 8 days of de-submergence in BC_3_F_3_**.

**Figure 9:**
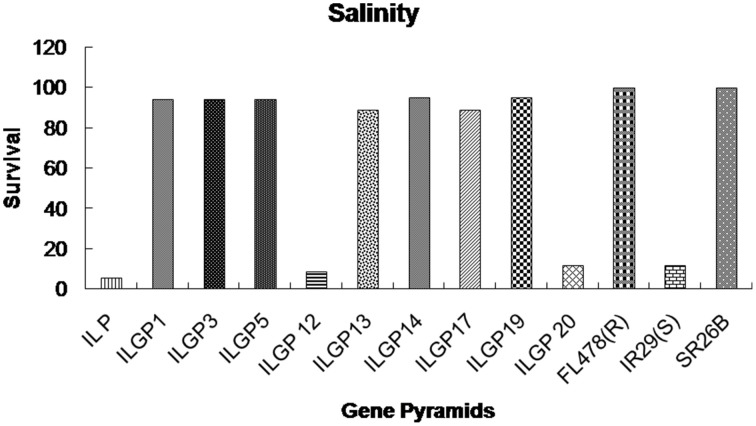
**Survival rates (%) of different Improved Lalat gene pyramid lines after salinity stress**.

**Figure 10:**
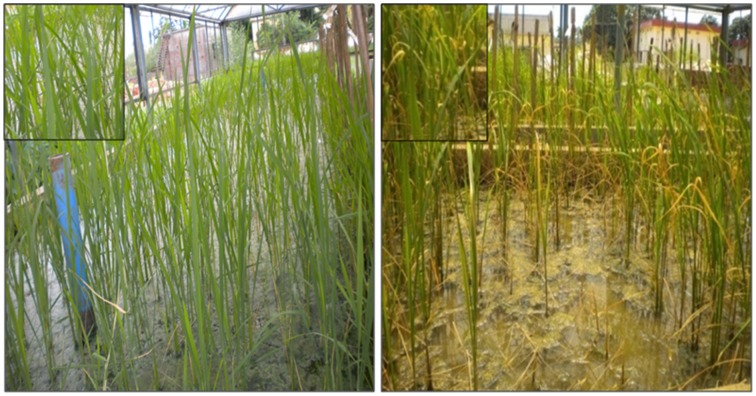
**Salinity screening of IL pyramid lines against salinity stress in BC_3_F_3_ generation**.

The genotype FR13A, a well-known donor for submergence tolerance, showed 100% regeneration after 15 days of submergence and 8 days of desubmergence while the recipient did not survive the submergence stress. Whereas the pyramid lines having *Sub1* QTL showed a different percentage of resistance and three lines showed nearly same as resistant control (Figures 7, 8). The line FL478, a MAS product having *saltol* transferred from Pokkali was found to be effective against salinity stress by showing 100% tolerance with the score 1 while the recipient parents showed 5.8% survival against the stress and the pyramid lines with *Saltol* QTL showed a high degree of resistance (Figures 9, 10).

The homozygous lines with different gene combination were subjected for field evaluation as the 10 gene combination plants showed enhanced resistance/tolerance to all the biotic and abiotic stresses in the bioassay and selected based on their morphology and grain quality characters for further evaluation in BC_3_F_3_ generation (Tables 5, 6).

**Table 5:** **Agronomic traits of the pyramids of Improved Lalat in BC_3_F_3_ generation**.

**Genotype**	**DFF**	**PH**	**PL**	**LL**	**LW**	**EBT**	**GL**	**GW**	**fertility%**	**1000 GW**
IL-P	106	99.66	24.0	34	1.2	13.0	9.27	2.43	84.0	22.76
ILGP1	108	92.00	24.0	25	1.1	12.2	8.19	2.20	79.9	23.62
ILGP3	101	99.00	27.0	41	1.2	11.0	9.32	2.38	80.3	27.00
ILGP5	106	89.00	25.0	34	1.5	12.3	8.12	2.76	80.0	25.05
ILGP12	101	93.00	32.0	30	1.1	13.3	9.06	2.23	81.2	19.50
ILGP13	102	89.30	28.0	41	1.0	14.4	9.46	2.23	78.9	16.00
ILGP14	101	98.00	30.0	38	1.2	11.3	9.71	2.45	83.3	27.00
ILGP17	102	96.30	21.0	27	1.0	14.0	8.69	2.41	82.4	20.00
ILGP19	110	93.60	26.0	33	1.4	12.0	8.98	2.21	82.0	23.89
ILGP20	98	92.00	25.0	27	1.1	11.3	9.41	2.27	84.2	17.00

*ILP, Improved Lalat Parent; ILGP, Improved Lalat gene pyramid; DFF, Days to 50% flowering; PH, Plant Height (in cm); PL, Penicle Length (in cm); LL, Leaf Length (in cm); LW, Leaf width (in cm); EBT, Ear bearing tiller (in no.); GL, Grain Length (in cm); GW, Grain Width (in cm); 1000 GW, 1000 Grain weight (in g)*.

**Table 6:** **Grain quality characteristics of grains of selected BC_3_F_3_ lines of Improved Lalat**.

**Genotype**	**Hulling (g)**	**Milling (g)**	**HRR (g)**	**KL (cm)**	**KB (cm)**	**L/B (cm)**	**ASV**	**VER (cm)**	**KLAC (cm)**	**Amylose (%)**
IL-P	79.0	72.0	62.0	6.86	2.57	2.70	4	3.75	10.60	23.85
ILGP1	80.0	75.0	63.0	6.57	2.59	2.55	4	3.75	10.30	20.62
ILGP3	76.0	72.0	62.0	6.8	2.54	2.69	4	3.75	10.00	21.11
ILGP5	78.0	69.5	62.5	5.67	2.91	2.00	4	3.75	9.70	20.47
ILGP12	75.5	69.8	59.5	6.67	2.33	2.87	4	3.75	10.30	23.88
ILGP13	78.0	73.5	63.5	6.52	2.35	2.79	4	3.75	10.40	21.03
ILGP14	77.0	71.0	54.5	6.99	2.42	2.89	6	4.00	10.80	22.72
ILGP17	76.0	71.0	55.5	6.46	2.52	2.56	4	3.75	10.60	21.07
ILGP19	80.0	74.5	63.5	6.72	2.61	2.58	4	3.75	11.00	23.77
ILGP20	80.0	72.5	57.5	6.46	2.53	2.56	4	3.75	10.60	23.85

*ILP, Improved Lalat Parent; ILGP, Improved Lalat gene pyramid; HRR, Head Rice Recovery (in g); KL, Kernel length (in cm); KB, Kernel breadth (in cm); KLAC, Kernel length after cooking (in cm); Hulling (in g); Milling (in g); ASV, Alkali spreading value*.

### Agro-morphological and quality characters of the improved lalat gene pyramids

The BC_3_F_3_ generation Improved Lalat gene pyramids were grown in the field for agronomic characterization and evaluation of their performance. The results indicate that the plant height of the lines ranged from 89 cm (ILGP5) to 99 cm (ILGP3), while 99.66 cm in (Improved Lalat) parent and in most of the recombinants, the plant height is nearly equal to the parent. The mean tiller (EBT) number varied from 11.0 to 14.4 and some lines showed slightly lower tiller number than the IL parent (13.0) and three lines showed the higher tiller number than the parent. One line ILGP17 (21) had shorter panicle length (PL) than parent Improved Lalat (24) while in all others; the values were nearer or slightly higher than the parent. The fertility percentage ranged from 78.9 to 84.2%. The 1000-grain weight was higher in ILGP3 and ILGP14 (27.00 g) and lowest in ILGP13 (16.00 g) (Table 5).

Most of the selections were found to be nearer to Improved Lalat (the recurrent parent), in grain quality. The rice kernel length varied from 5.67 to 6.99 cm and the L/B ratio value of most of the lines is in the range of 2.55–2.89 cm except ILGP5 (2.00 cm) while parent Improved Lalat has an L/B ratio of 2.70 cm. Two lines, ILGP13 and ILGP19 (63.50 g) showed the higher HRR value in comparison to Improved Lalat (62.00 g). With respect to ASV, maximum lines are similar to their parental value (4) one line LGP 14 has a slightly higher value (6). The KLAC values in the lines ranged from 9.70 to 11.00 cm and some are better than the Improved Lalat parent (10.60 cm) while one line slightly lesser with 9.70 cm. All Improved lines had amylose content in the range of (20.47–23.88%) and the values are nearer to that of the Improved Lalat parent (23.85%) (Table 6).

### Improved lalat -SSR based background selection

Genetic similarity analysis on 9 pyramided lines of Improved Lalat, two lines with (*Gm1* + *Gm4* + *Pi2* + *Pi9* + *Sub1* + *Saltol* + *Xa21* + *xa13* + *xa5* + *Xa4*), one line with (*Gm1* + *Gm4* + *Sub1* + *Saltol* + *Pi2* + *Xa21* + *xa13* + *xa5* + *Xa4*), three with (*Gm1* + *Gm4* + *Sub1* + *Saltol* + *Xa21* + *xa13* + *xa5* + *Xa4*), one line with (*Gm1* + *Gm4* + *Pi2* + *Saltol* + *Xa21* + *xa13* + *xa5* + *Xa4*), one line with (*Sub1* + *Saltol* + *Xa21* + *xa13* + *xa5* + *Xa4*) and one with (*Pi2* + *Pi9* + *Xa21* + *xa13* + *xa5* + *Xa4*), and Improved Lalat parent was performed with 45 SSR markers with an objective to select the lines that had higher number of alleles of the recurrent parent.

#### Number of alleles amplified, allele size, and PIC value

The Improved Lalat and the six donor parents were tested with 600 SSR primers which were spread across all the 12 chromosomes of rice. Out of them, 45 primers were selected which were polymorphic between the parent and donors that 45 primers were used for background, selection of Improved Lalat and its 9 pyramided lines with different gene combinations.

A total of 61 reproducible bands was obtained from 45 microsatellite markers (SSRs). The number of alleles varied from 1 to 3. The size of the amplified fragments within a range of 50 bp (RM302) to 750 bp (RM440). Most of the fragments varied from 100 to 300 bp. The mean PIC value of all polymorphic primers was 0.07. The maximum numbers of alleles (3) were amplified with three primers RM447, RM4838, and RM490. Ten primers produced 2 alleles whereas maximum primers were produced a single allele. The highest PIC value was shown in RM4838 (0.73) while the lowest PIC value was seen in RM2144 (0.19) (Table 7).

**Table 7:** **Number of alleles and PIC values of 45 SSR markers used for background selection**.

**Sl. No**.	**Locus**	**Ch. No**.	**No of bands**	**PIC value**
1	RM286	11	2	0.59
2	RM302	1	1	0.00
3	RM171	10	1	0.00
4	RM565	3	1	0.00
5	RM424	2	2	0.00
6	RM138	2	1	0.00
7	RM324	2	1	0.00
8	RM232	3	1	0.00
9	RM263	2	2	0.00
10	RM129	1	1	0.00
11	RM496	10	2	0.00
12	RM447	8	3	0.00
13	RM437	5	1	0.00
14	RM440	5	1	0.00
15	RM3642	1	1	0.00
16	RM3195	3	2	0.00
17	RM1248	5	1	0.00
18	RM1359	4	1	0.00
19	RM1155	4	2	0.71
20	RM3337	4	1	0.00
21	RM4838	5	3	0.73
22	RM3805	6	1	0.00
23	RM3583	7	1	0.00
24	RM5711	7	1	0.00
25	RM3859	7	2	0.71
26	RM6369	8	1	0.00
27	RM2144	9	1	0.19
28	RM401	4	1	0.00
29	RM495	1	2	0.00
30	RM490	1	3	0.00
31	RM475	2	1	0.00
32	RM482	2	1	0.00
33	RM570	3	1	0.00
34	RM418	7	1	0.00
35	RM442	3	1	0.00
36	RM204	6	1	0.00
37	RM324	2	1	0.00
38	RM224	11	1	0.00
39	RM472	1	1	0.00
40	RM587	6	2	0.00
41	RM585	6	1	0.00
42	RM314	6	1	0.00
43	RM287	11	1	0.00
44	RM279	2	1	0.00
45	RM423	2	2	0.00

#### Genetic similarity using SSR data

Using the data obtained on the background, selection of Improved Lalat and the gene pyramids, genetic similarities were calculated and the genetic similarity between the pyramid lines and Improved Lalat parent (IL P) had an average value of 0.98. Three pyramid lines ILGP3, ILGP19, and ILGP20 showed highest similarity with parent (1.00). ILGP12 showed 0.99 similarities with parent. Three pyramid lines, ILGP1, ILGP5, and ILGP13 showed 0.98 similarities with parent and ILGP14 showed 0.96 similarities while ILGP17 showed the least similarity with a parent having a value of 0.94.

##### Cluster analysis

**Figure 11:**
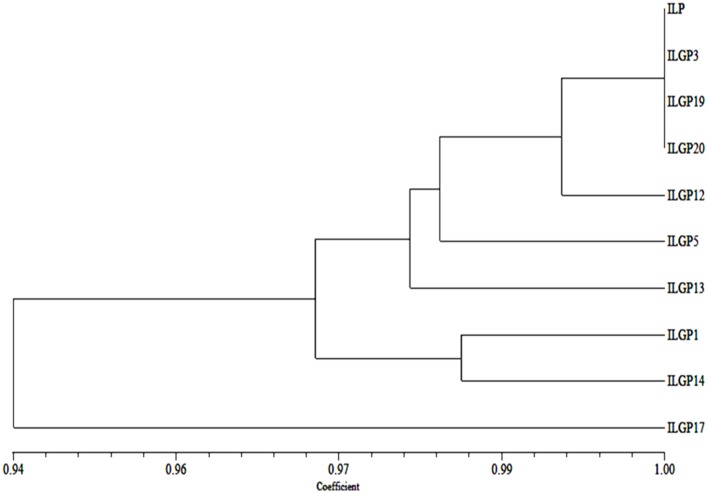
**Dendogram generated based on SSR data on pyramid lines of Improved Lalat**.

The dendrogram clearly explains the relationships among the pyramid lines with their parent. Dice genetic similarity index was used to study the genetic relationship toward parents, and based on the dendrogram generated, 9 genotypes, including Improved Lalat parent (IL P) were grouped into two major clusters. Cluster I consisted of ILGP17. The cluster II consisted of all the pyramid lines and Improved Lalat parent (IL P). Cluster II was further grouped into two sub clusters. Cluster II-A and cluster II-B. Cluster II-A was consisted of ILGP1 and ILGP14 while cluster II-B consisted of Improved Lalat parent with rest pyramid lines. Cluster II-B was again divided into two sub clusters, cluster II-B-i and II-B-ii. Cluster II-B-i was again divided into two sub clusters. Cluster II-B-i-a was having ILGP13 while another cluster II-B-i-b was again divided into two sub clusters, one was having ILGP5 while another was again divided into two groups, one was having ILGP12 while in another one ILGP20, ILGP19, ILGP3, and IL P were closer to each other by forming a single line (Figure 11).

### Genetic distance and cluster analysis of improved lalat based on combining both morphological and quality traits

#### Distance matrix

Combining values of both agronomic and quality traits, genetic distance of 9 pyramid lines were calculated between each pair of observations. The average genetic distance of IL P to all pyramid lines was with a value of 0.27% distance. The result of pair wise comparisons indicated that ILGP17 was closer (0.23) distance to Improved Lalat parent (IL P) than any other with value of distance. While ILGP3, ILGP14, ILGP5, and ILGP19 were closer to parent with 0.28 and 0.29% distance. ILGP20 was closer to IL parent with 0.31 distance. ILGP1 and ILGP12 were 0.33 and 0.34% distant from the parent. ILGP 13 was seen to be the maximum distance from the IL parent.

#### Cluster analysis

**Figure 12:**
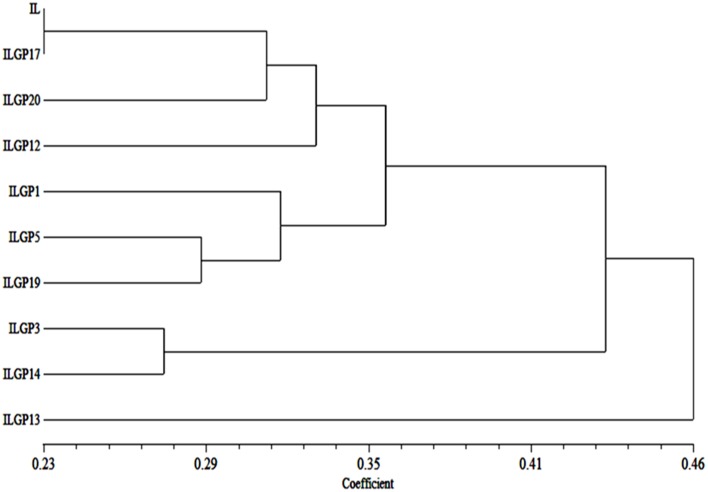
**Dendogram generated based on combining both morphological and quality data on pyramid lines of Improved Lalat**.

A genetic distance of Improved Lalat (IL P) and 9 pyramid lines were produced a dendogram (Cluster tree analysis) which clearly explains the relationships among all the pyramid lines with their parent. There are two major clusters, cluster I and cluster II. The cluster I was having ILGP13 and the cluster II was having IL P with 8 pyramid lines. Cluster II was divided into two subgroups, i.e., cluster IIA and cluster IIB. Cluster IIA was having ILGP13 and ILGP14, while cluster IIB was again divided into two subgroups, i.e., cluster IIB i and cluster IIB ii. Cluster IIB i was again divided into two groups, one was having ILGP1 and another was having ILGP5 and ILGP19 which formed one cluster. Cluster IIB ii was again divided into two groups. One was having ILGP12 and another one was again divided into two groups, one was having ILGP20 and another one formed a single line consisted of IL P and ILGP17 (Figure 12).

## Discussion

The tightly linked molecular markers for economically significant traits have been identified and used for marker assisted backcross breeding in rice including resistance to biotic stresses BB, blast, gall midge, and abiotic stresses submergence and salinity additionally to some other stresses (Hasan et al., 2015). Marker-assisted gene pyramiding of key genes/QTLs has helped in tacking susceptibility for foremost diseases and insects such as bacterial blight (Huang et al., 1997; Singh et al., 2001; Suh et al., 2011), blast (Hittalmani et al., 2000; Singh et al., 2011, 2012; Zhou et al., 2011) and gall midge (Katiyar et al., 2001; Singh et al., 2014b) etc. In India, enhancement of broad scale resistance against BB is a foremost challenge due to the high diversity in agro climatic zones where rice is cultured along with the existence of a numeral of genetically distinct virulent *Xoo* strains in diverse environmental areas of India. Deployment of a combination of genes can attain strong and broad range resistance in many BB prone rice growing areas (Dokku et al., 2013a,b; Pradhan et al., 2015). Marker assisted foreground selection was also used for effective stacking of abiotic stress traits such as submergence tolerance (Neeraja et al., 2007; Iftekharuddaula et al., 2011; Khanh et al., 2013; Divya et al., 2014) and salinity tolerance (Thomson et al., 2010; Moniruzzaman et al., 2012; Divya et al., 2014).

Due to environmental stress or abiotic stress, if rice plants stay submerged for more than 5 days, they start to die and there is almost no chance to recover after the water recedes. The International Rice Research Institute (IRRI) determined to identify and introduced various submergence-tolerant rice genotypes since 1970s (Vergara and Mazaredo, 1975). In the 1970s, FR13A was identified as one of the best submergence-tolerant donors and was used extensively by rice breeders. Especially in coastal areas where floodwaters are often saline, the pyramiding of submergence and salinity tolerance is very important. Recently the major salinity QTL has been identified on chromosome 1 and characterized (Thomson et al., 2010). Though, numerous QTLs are possibly essential to attain adequate salinity tolerance in the field, also extra QTLs for seedling and reproductive-stage salinity tolerance may be required to afford defense from salinity stress during the rainy season. The prospect to apply molecular marker technologies as a means of stacking numerous tolerance genes/QTLs into single rice varieties delivers a distinctive opportunity for breeders to develop tolerant cultivars more rapidly for targeted environments (Septiningsih et al., 2013) and different types of biotic stresses. The *Sub1* gene positioned on rice chromosome 9 was well-known as a foremost gene conferring submergence tolerance in tolerant rice cultivar FR13A and its derived progenies (Xu and Mackill, 1996; Xu et al., 2000, 2004; Toojinda et al., 2003; Manivong et al., 2014).

In this study, the improvement of resistance against blast, gall midge including bacterial blight disease and tolerable to submergence and salinity stress with effective foreground and SSR based background selection was achieved by following three back crosses and three generation selfing, resulting the high level of resistance or tolerance to biotic and abiotic stresses. Similar results were reported with gene pyramiding in different rice varieties with different resistance genes for biotic and abiotic stresses (Sanchez et al., 2000; Singh et al., 2001; Dokku et al., 2013a,b; Gouda et al., 2013; Suh et al., 2013; Divya et al., 2014; Kumar et al., 2014; Pradhan et al., 2015).

The study employed three backcrosses to transfer the desired traits from the donors into Improved Lalat followed by three cycles of selfing (Figure 1). Through this approach, we could effectively transfer all the target genes/QTLs of resistance/tolerance into the Improved Lalat background using marker assisted selection in each step starting from BC_1_F_1_ generation (Figure 1). Clear polymorphism was observed with all the markers linked to the genes/QTLs under the study between the parent and the donors (Figure 2). Of the two hundred fifty BC_3_F_3_ generation lines of Improved Lalat, 31 plants homozygous for alleles associated with the resistance/tolerance with the gene combination were selected (Figure 1) through marker analysis. Nine lines with the presence and absence of targeted genes/QTLs were selected with different gene combinations using tightly linked markers (Figures 3, 4). The linked markers used in this study were, according to the published literature (Table 1). Similar results were obtained in rice using the same strategy for BB resistance by several workers, including our laboratory at CRRI earlier (Dokku et al., 2013a,b; Pradhan et al., 2015). The recovery of the entire target genes/QTLs suggested that the population size employed in the study was enough to recover the desired combinations. However, with higher population levels, higher number of gene pyramids with varied content of the recurrent parental genome could have been obtained and selection options would have been higher. This is evident from the background analysis as in some of the lines; the recovery of the recurrent parent genome was not up to the expected level.

The purpose of the program is to fortify the plant defenses without disturbing the recurrent parental genome through marker assisted approach so that the new gene pyramid will be acceptable to the farmers, millers and traders. For ensuring the acceptability of the new genotypes, it is necessary to characterize the gene pyramids and select the ones that are closer to the parent for both morphological and quality traits as this selection add value to the MAS program. These genetic similarity studies conclusively prove the utility of both the molecular and morphological analysis in efficient background selection.

The bioassays confirmed the earlier finding that the recurrent parent possess high levels of resistance against BB and all the pyramids developed had all the BB resistance genes and all of them showed high levels of resistance (Table 2). The recurrent parent along with pyramids of different gene combinations including two gene pyramids having 10 genes that showed a high degree of resistance against the most virulent BB isolates with lesion length below 3.4 cm. Whereas, ILGP1, ILGP14 showed the highest resistance with less than 0.9 cm lesion length (Table 2) against BB similar with the previously reported results (Rajpurohit et al., 2011; Dokku et al., 2013a,b; Suh et al., 2013; Pradhan et al., 2015).

Pyramiding multiple resistance genes in case of blast disease is a necessity as a breakdown of resistance is known against blast disease and of the several available genes that confer resistance against blast, a combination of *Pi2* and *Pi9* (the gene from *O. minuta*, a wild species of *Oryza* family) was considered to be ideal for dealing with blast (Hittalmani et al., 2000; Liu et al., 2002; Qu et al., 2006). In case of Blast in the current study, the pyramids which were having genes *Pi2* and *Pi9* were screened for the blast in the research institute at Hazaribaag India, and these lines showed a score of “0,” a resistant reaction while some lines were moderately resistant (MR) with a score “3.” It was evident from the results that the gene combination *Pi2+Pi9* were more effective (ILGP5, ILGP17, ILGP19) by showing resistant reaction while the lines with either *Pi2* or *Pi9* alone were susceptible or moderately resistant to Blast and the recurrent parent had shown highly susceptible reaction (Table 3, Figure 5). The genetic enhancement of the genotype, Improved Lalat in terms of resistance to blast in a hot spot region confirms the utility of *Pi2*+*Pi9* gene combination in eastern India against blast.

Gall midge is a major insect pest on rice (Behura et al., 2000). Several biotypes are known and recent breakdown of resistance in different parts of the country is causing fear. There are 11 gall midge resistance genes reported so far. A combination of *Gm1* and *Gm4* genes was considered to be ideal to confer resistance against this major pest (Nair et al., 1996; Biradar et al., 2004). In our study, the gene pyramids having the *Gm1*+*Gm4* gene combination expressed a high degree of resistance against gall midge, the levels that are comparable to that of the donor parents. In Improved Lalat genotype, the pyramids which were having both genes *Gm1* and *Gm4* when screened for their reaction against Gall midge, showed high levels of tolerance against gall midge amongst, ILGP5 and ILGP14 showed the highest degree of resistance with 100 and 94.1% positive plants respectively (Table 4, Figure 6). The present result suggested the utility of this gene combination in the combating gall midge, major insect pest affecting rice cultivation.

Among the abiotic stresses in rice, salinity and submergence are the two major stresses that cause severe yield losses year after year. In the changing scenario of climate change, they assume greater importance. With the rising of sea levels, more land area is likely to get inundated and with increasing precipitation, submergence of standing crops is a reality.

With the recent identification of major QTLS like *Sub 1* (Xu et al., 2006) and *Saltol* (Bonilla et al., 2002; Nejad et al., 2008, 2010) conferring tolerance against submergence and salinity respectively is feasible.

With the development of Swarna Sub 1 through the transfer of *Sub 1* QTL from FR 13A, the utility of the *Sub1* QTL was well demonstrated at IRRI, Philippines. The present study also strengthens the utility of *Sub1* QTL as the pyramids having the *Sub1* did exhibit the expected levels of tolerance to submergence. Among them ILGP5, ILGP19 and ILGP20 showed the highest degree of submergence and the tolerance levels are on par with the level exhibited by FR13A (Figures 7, 8).

The *Saltol* QTL was transferred from Pokkali into different genotypes like FL478 that showed high promise in earlier studies at CRRI, India. The lines having *Saltol* QTL when screened for tolerance against salinity stress, survived the stress with minor variations in degree of tolerance, but scoring was between 1 and 3 on a scale of 0–9 suggesting a high level of tolerance in several gene pyramids (ILGP1, ILGP3, ILGP5, ILGP13, ILGP14, ILGP17, and ILGP19) while the recurrent parent (Improved Lalat) was susceptible with a score of 9 and did not survive the salinity stress (Figures 9, 10).

The results indicated that the genes in combination were more effective than a single gene and significant genetic enhancement was observed in Improved Lalat the recurrent parent, for all the target traits that were transferred in the present study. However, despite achieving a high level of success in both gene transfer and their expression in several gene pyramids, some of the pyramids did not exhibit the desired levels of expression of tolerance/resistance against the stresses suggesting the role of the genetic background in showing the level of expression. Another possibility for this deviation could be a result of recombination between the marker locus and the respective gene conferring resistance if the marker and the gene/QTL are quite distant. Therefore, there is a need to develop a suitable PCR based marker that is physically closer to the target gene and use of gene based markers is the best solution for effective transfer and expression of the transferred gene at the desired level.

In the current study, we employed three backcrosses followed by selfing to derive the maximum genetic background of Improved Lalat and the BB resistance genes. The SSR based background selection was employed on the pyramids in BC_3_F_3_ generation that revealed the recovery of more than 90% of the recurrent parent alleles (Figure 11) which corroborates with previous researches that employed different markers like AFLP (Chen et al., 2001) and RFLP (Chen et al., 2000). At a single locus, the expected frequencies for individuals to be homozygous for the genotype of the recurrent parent would be 0.5, 0.75, and 0.875, in BC_1_F_1_, BC_2_F_1_, and BC_3_F_1_ respectively. These can also be viewed as the expected proportions of loci for individuals to be homozygous for the recurrent parent genotype. The variances of such proportions in these generations would be 0.5 (1 2 0.5)/*n*, 0.75 (1 2 0.75)/*n*, and 0.875 (1 2 0.875)/*n*, respectively, where *n* is the number of independent recombination units in the genome. Though it is difficult to match the map units that are equivalent to an independent recombination unit in the rice genome, it is clear that the variance in BC_1_F_1_ is much larger than those in subsequent generations, indicating a wider frequency distribution in the BC_1_F_1_ than the later generations. The desired individual with recombination between the targeted gene locus and either one of the flanking markers is expected to occur at a much higher frequency in BC_1_F_1_ than BC_2_F_1_ which means that it is feasible to practice background selection in the BC_1_F_1_ generation. Thus, in addition to the background selection in the BC_3_F_1_ generation, adding one more round of background selection in BC_1_F_1_ to the MAS scheme may greatly increase the efficiency of the program and selection for one more cycle for morphological and grain quality traits may result in identification of lines that are much more closer to their parent. In this study the pyramids also evaluated for the intimacy of pyramids for its agro morphological and grain quality traits with the recurrent parent. The dendogram combining both morphological and grain qualities showed that the selected nine pyramids were closest to the recurrent parent with less distance to parent (Figure 12).

The current work is aimed to combine multiple genes/QTLs into an elite rice variety Improved Lalat for enhanced resistance/tolerance against several stresses. The results suggested that, the pyramidization of 10 genes into an elite variety have significantly enhanced the level of resistance/tolerance against the target stresses for which they are known to be associated. In addition, with the selection at the morphological level, grain quality and SSR based selection, we could recover most of the recurrent parental genome, which is clear from the dendrograms and some of the gene pyramids are closer to their respective recurrent parents in many morphological and quality traits. Thus, the study has achieved the major objective through successful transfer of six important genes/QTLs to confer resistance/tolerance against the major biotic and abiotic stresses and the four BB resistance genes present in the recurrent parent also displayed a higher level of resistance in the bioassays. Two gene pyramids like (ILGP5, ILGP19) that have all the 10 genes have shown great promise and have the potential with resistance/tolerance against multiple stresses. Identification of genes with similar reactions to two or more races is difficult to identify in conventional breeding making transfer difficult through conventional approaches. However, with the availability of molecular markers that are closely linked with each of the target genes make the identification of plants with more than two genes possible enabling us to incorporate multiple desirable genes into a single elite genotype. The success of the work clearly demonstrates the probability of addressing the problems of bacterial blight, blast, gall midge along with submergence and salinity stresses which is a major challenge to the rice productivity.

## Conclusion

Development of broad-spectrum resistance against diseases like BB and blast and insects like gall midge in the Indian subcontinent is a foremost challenge due to the rich multiplicity of the agro-climatic zones where rice is cultured, as well as the presence of a number of genetically distinct virulent strains/biotypes in different geographical areas of India. In addition, in the climate change scenario, tolerance to submergence, and salinity assume great significance to rice particularly in the rain fed ecologies. The study has demonstrated that deployment of appropriate gene or gene combinations against each stress can achieve durable and broad-spectrum resistance/tolerance in stress prone specific areas of India. The success may also stimulate several such studies to realize the potential of molecular plant breeding as the foundation for crop improvement in the twenty-first century.

The future studies planned on these gene pyramids include evaluation against the target trait on a larger scale in a multilocation environment so as to determine the expression of the incorporated genes/QTLs. These studies can provide us additional information on the interaction between the incorporated genes, if any. In addition, with a further selection at both morphological and molecular levels in large populations of these gene pyramids, we wish to recover the recurrent parental genome in full and NILs of each genotype can be established with each one of the nine genes/QTLs. These NILs will be of great use in the future rice breeding programs and breeders can use them suiting to their needs either individually or in desired combinations.

### Conflict of interest statement

The authors declare that the research was conducted in the absence of any commercial or financial relationships that could be construed as a potential conflict of interest.

## Footnotes

^1^Annual Report 2011-12. Central Rice Research Institute, Cuttack, p 21.
